# Metabolic analysis of the loss of *Rb1 in vivo*

**DOI:** 10.1186/2049-3002-2-S1-O4

**Published:** 2014-05-28

**Authors:** Brandon N Nicolay, Paul S Danielian, Wilhelm Haas, Gregory Stephanopoulos, Jacqueline A Lees, Nicholas J Dyson

**Affiliations:** 1 Massachusetts General Hospital Cancer Center, Harvard Medical School, Charlestown, MA, USA; 2 David H. Koch Institute for Integrative Cancer Research, MIT, Cambridge, MA, USA; 3 Massachusetts Institute of Technology, Cambridge, MA, USA

## 

Inactivation of the p16/pRB axis is a rate-limiting step in tumorigenesis. Loss of these tumor suppressors is well known to disrupt control of cell proliferation but, additionally, these mutations cause changes in cell metabolism. To investigate the metabolic changes resulting from pRB inactivation we have exploited a Drosophila model. Metabolomics and *in vivo* flux analysis revealed that RBF-deficient tissues have increased nucleotide production and struggle to maintain sufficient pools of reduced glutathione; ultimately leading to increased sensitivity to oxidative stress. We have now extended these studies to mammalian cell culture and mice. Depletion of pRB in non-tumorigenic hTERT-BJ cells leads to an increased cell population in S-phase and slight increases in the cell population doubling time. Acute loss of *Rb1-/-*in the mouse colon, but not the lung, leads to a robust increase in Ki67 staining within 96hrs. We therefore asked to what extent metabolic changes associated with proliferation increases are conserved between pRB-deficient cells in culture and acute loss of *Rb1-/-in vivo*. We performed isotopic tracer analyses with both glucose and glutamine and found that while loss of pRB in cell culture promotes increased isotopic enrichment from U13C-glutamine in TCA cycle intermediates, little to no change was seen in isotopic enrichment from U13C-glucose. In stark contrast, acute loss of *Rb1* in the mouse colon leads to robust isotopic enrichment from U13C-glucose in TCA cycle intermediates and the pyrimidine precursor aspartate; while little to no change in isotopic enrichment from U13C-glutamine was found in all TCA cycle intermediates but alpha-ketoglutarate and no significant differences was seen in aspartate. Collectively these results reveal that loss of the tumor suppressor pRB has metabolic consequences *in vivo*.

